# Cloning, expression, purification, and structural modeling of the Chandipura virus matrix protein

**DOI:** 10.1002/2211-5463.70130

**Published:** 2025-09-25

**Authors:** Mariana Grieben

**Affiliations:** ^1^ Institute of Biochemistry, Center of Structural and Cell Biology in Medicine University of Lübeck Germany

**Keywords:** Chandipura virus, Chandipura virus matrix protein, cloning, encephalitis, expression, purification, Vesiculovirus

## Abstract

The Chandipura virus matrix protein plays a crucial role in virus assembly, budding, and the cytopathic effects observed in infected cells by interacting with several host proteins. The protocol presented here outlines the expression and purification of full‐length Chandipura virus matrix protein and two N‐terminally truncated constructs produced in *Escherichia coli*. This protocol results in high yields of monomeric matrix protein, which is suitable for structural studies. Additionally, GFP‐fused Chandipura virus matrix protein constructs can be expressed in mammalian cells for examination of intracellular localization. The Chandipura virus matrix protein model, generated using AlphaFold, features an intrinsically disordered N terminus and a structured C‐terminal core, similar to other Vesiculovirus matrix proteins.

## Graphical abstract

This research protocol offers a guide for the cloning, expression, and purification of the Chandipura virus matrix protein using *E. coli*. It also includes a step‐by‐step procedure for cloning, expressing, and conducting fluorescence imaging of GFP‐fused Chandipura virus matrix protein in mammalian cell lines. This study aims to facilitate interaction studies between the Chandipura virus matrix protein and human proteins.

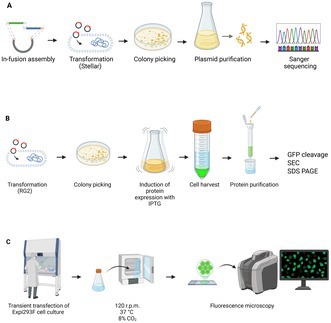

**Abbreviations**
BLAST: basic local alignment search tool
CHPV: Chandipura virus
CHPV M: Chandipura virus matrix protein
DDM: n‐dodecyl‐β‐D‐maltoside
DTT: dithiothreitol
*E. coli*
: *Escherichia coli*
ELISA: enzyme‐linked immunosorbent assay
GFP: green fluorescent protein
GST pull‐down: glutathione S‐transferase pull‐down
HEPES: 4‐(2‐hydroxyethyl)‐1‐piperazineethanesulfonic acid
IMAC: immobilized metal affinity chromatography
IPTG: isopropyl β‐d‐1‐thiogalactopyranoside
LB: Luria‐Bertani
MW: molecular weight
pLDDT: predicted local distance difference test
SDS/PAGE: sodium dodecyl‐sulfate polyacrylamide gel electrophoresis
Single‐particle cryo‐EM: single‐particle cryo‐electron microscopy
TB: Terrific Broth
TCEP: tris(2‐carboxyethyl)phosphine
TEV: Tobacco etch virus
VSIV M: Vesicular stomatitis Indiana virus matrix protein
VSIV: Vesicular stomatitis Indiana virus
VSV M: Vesicular stomatitis virus matrix protein
VSV: Vesicular stomatitis virus
ε: molecular extinction coefficient

Chandipura virus (CHPV) is a virus that belongs to the genus Vesiculovirus in the family Rhabdoviridae. It is an emerging human pathogen that causes deadly encephalitis outbreaks in children in India. The virus enters the central nervous system by crossing the blood–brain barrier, mainly targeting neurons [1, 2, 3, 4]. The infection starts with flu‐like symptoms and develops into acute encephalitis, leading to coma and death in up to 75% of cases [5]. CHPV is transmitted by vectors such as mosquitoes, ticks, and sandflies [6]. Despite the high mortality rates, this virus remains poorly studied, and unfortunately, no treatment is currently available for this emerging human pathogen [7].

The 11 kb negative‐sense single‐stranded RNA genome encodes the glycoprotein (G), the matrix protein (M), the nucleoprotein (N), the phosphoprotein (P), and the large polymerase protein (L) through five different monocistronic mRNAs, in sequential order and decreasing amounts [8, 9]. Expression and purification of the M, G, N, and P proteins of CHPV were first reported by Kumar and co‐workers [10] to study the interaction between them. These protocols have been developed to conduct ELISA and GST pull‐down assays, rather than structural studies.

The Chandipura virus matrix protein (CHPV M), one of the five viral proteins, has been identified as a pathogenic protein [8]. Most of our current understanding of CHPV M comes from studies of the M proteins of closely related vesiculoviruses. These studies suggest that this protein is critical in virus assembly and budding, apart from its structural role. Additionally, it is predicted to be responsible for most of the cytopathic effects observed in infected cells, such as cell rounding and apoptosis [11], by interacting with its host in various ways [9]. However, these interactions have not been studied so far.

CHPV M is a multifunctional protein that is 229 amino acids long. The N‐terminal tail of the M protein is predicted to be structurally disordered (residues 1–30). The seven lysine residues within the first twenty N‐terminal residues are believed to facilitate the binding of the protein to membranes as well as promote self‐association of CHPV M. The N‐terminal region also includes a PPSY sequence (30–33) [11]. PPxY motives (x denotes any amino acid), found in the matrix proteins of various viruses, are crucial for the interaction with host cell proteins containing WW domains [12]. Most of the protein is predicted to adopt a secondary structure (residues 30–229) [11]. However, no molecular structure of CHPV M is available to date.

CHPV M contributes to CHPV‐induced encephalitis by interacting with host proteins in neurons. In this study, constructs for expressing CHPV M in bacterial cells were cloned, and a protocol for producing high yields of monomeric full‐length CHPV M and two N‐terminal truncated variants from *E. coli* was established. Additionally, constructs for the expression of full‐length CHPV M in mammalian cells will facilitate intracellular localization and co‐localization studies using fluorescence microscopy.

## Materials

### Cloning and plasmid purification

1. Synthetic CHPV M gene purchased from Twist Bioscience (UniProtKB Q9WH76).
2. pET28a(+) (Merck‐Millipore #69864) and pcDNA3.1 (Thermo Fisher #V79020) vectors.
3. Superfolder GFP [13].
4. In‐Fusion Snap Assembly cloning kit and Stellar chemically competent cells (Takara Bio #638945).
5. Thermocycler.
6. Agarose Gel Electrophoresis.
7. Gel and PCR Clean‐up kit (Macherey‐Nagel #740609).
8. 42 °C water bath.
9. Sterile 1.5 and 15 mL centrifuge tubes.
10. LB (Luria‐Bertani) liquid medium: 10 g tryptone, 5 g NaCl, 5 g yeast extract in 1 L ddH_2_O, and pH 7.2–7.5 (autoclave for 15 min at 120 °C).
11. LB agar (Luria–Bertani broth with 1.5% agar–agar) plates supplemented with antibiotics.
12. Antibiotics: ampicillin (BioChemica #A0839) and kanamycin (Gerbu #1091).
13. Glass Erlenmeyer flasks.
14. Laboratory shaker with thermoregulation.
15. Refrigerated centrifuge and centrifuge tubes.
16. NucleoSpin Plasmid kit (Macherey‐Nagel #740588).
17. NucleoBond Xtra Midi EF kit (Macherey‐Nagel #740420).
18. Spectrophotometer (DeNovix DS‐11).

### Protein expression in *E. coli*

1. Rosetta‐gami™ 2(DE3) (Merck‐Millipore #71351).
2. LB agar plates supplemented with antibiotics.
3. TB (Terrific Broth) liquid medium: 12 g tryptone, 24 g yeast extract, 4 mL glycerol, 2.313 g potassium dihydrogen phosphate, 12.54 g potassium phosphate dibasic in 1 L ddH_2_O (autoclave for 15 min at 120 °C).
4. Antibiotics: kanamycin and chloramphenicol (SERVA #16785).
5. Glass Erlenmeyer flasks.
6. Laboratory shaker with thermoregulation.
7. Isopropyl β‐d‐1‐thiogalactopyranoside (IPTG) (Gerbu #1043).
8. Refrigerated centrifuge and centrifuge tubes.

### Protein expression in Expi293F

1. Laminar flow cabinet.
2. One vial of cryopreserved Expi293F mammalian cells (Thermo Fisher #A14527).
3. FreeStyle™ 293 Expression Medium (Thermo Fisher #12338018).
4. 37 °C water bath.
5. Corning^®^ Erlenmeyer cell culture flasks (Merck‐Millipore #CLS431143).
6. Sterile serological pipettes.
7. Sterile and filtered pipette tips.
8. Sterile 1.5, 2, 15, and 50 mL centrifuge tubes.
9. ExpiFectamine 293 transfection kit (Thermo Fisher #A14525).
10. PEI STAR™ transfection reagent (Tocris #7854).
11. CO_2_ orbital shaker with thermoregulation.
12. Automated cell counter (Countess II, Invitrogen).

### Protein purification

1. Continuous flow cell disruptor (Constant Systems).
2. Syringes and PES Membrane Filters (SARSTEDT #83.1826.001) for sterile filtration of buffers.
3. Sterile 1.5, 2, 15, and 50 mL centrifuge tubes.
4. Tobacco etch virus (TEV) protease [14].
5. Buffer A: 50 mm HEPES pH 7.5, 500 mm NaCl, 5% glycerol, Roche cOmplete EDTA‐free protease inhibitor cocktail, 100 μg·mL^−1^ DNAse I.
6. Buffer B: 50 mm HEPES pH 7.5, 500 mm NaCl, 5% glycerol, 5 mm imidazole.
7. Buffer C: 50 mm HEPES pH 7.5, 500 mm NaCl, 5% glycerol, 500 mm imidazole.
8. Buffer D: 50 mm HEPES pH 7.5, 500 mm NaCl, 5% glycerol.
9. Buffer E: 20 mm HEPES pH 7.5, 200 mm NaCl.
10. n‐dodecyl‐ß‐D‐maltoside (DDM) (Carl Roth #CN26.1).
11. Refrigerated centrifuge and centrifuge tubes.
12. Empty chromatography column.
13. TALON^®^ Metal Affinity Resin (Takara Bio #635502).
14. PD MidiTrap™ G‐25 columns (Merck‐Millipore #28918008).
15. Amicon^®^ Ultra Centrifugal Filter, 10 kDa MWCO (Merck‐Millipore #UFC5010).
16. Superdex^®^ 200 Prep Grade (Merck‐Millipore #GE17‐1043‐01).
17. Spectrophotometer (DeNovix DS‐11).
18. SDS/PAGE apparatus, 15% SDS/PAGE gels, 5× SDS/PAGE sample loading buffer, and Coomassie Brilliant Blue staining solution.
19. PageRuler™ Plus Prestained Protein Ladder (Thermo Scientific™ #26619).
20. Liquid nitrogen.

### Thermal shift assay

1. MicroAmp Fast Reaction tubes and caps (Thermo Fisher #4358293 and #4323032).
2. Refrigerated centrifuge.
3. SYPRO^®^ Orange Protein Gel Stain (Merck‐Millipore #S5692).
4. Applied Biosystems real‐time PCR system.

## Methods

### CHPV M 3D‐structure prediction

**Fig. 1:**
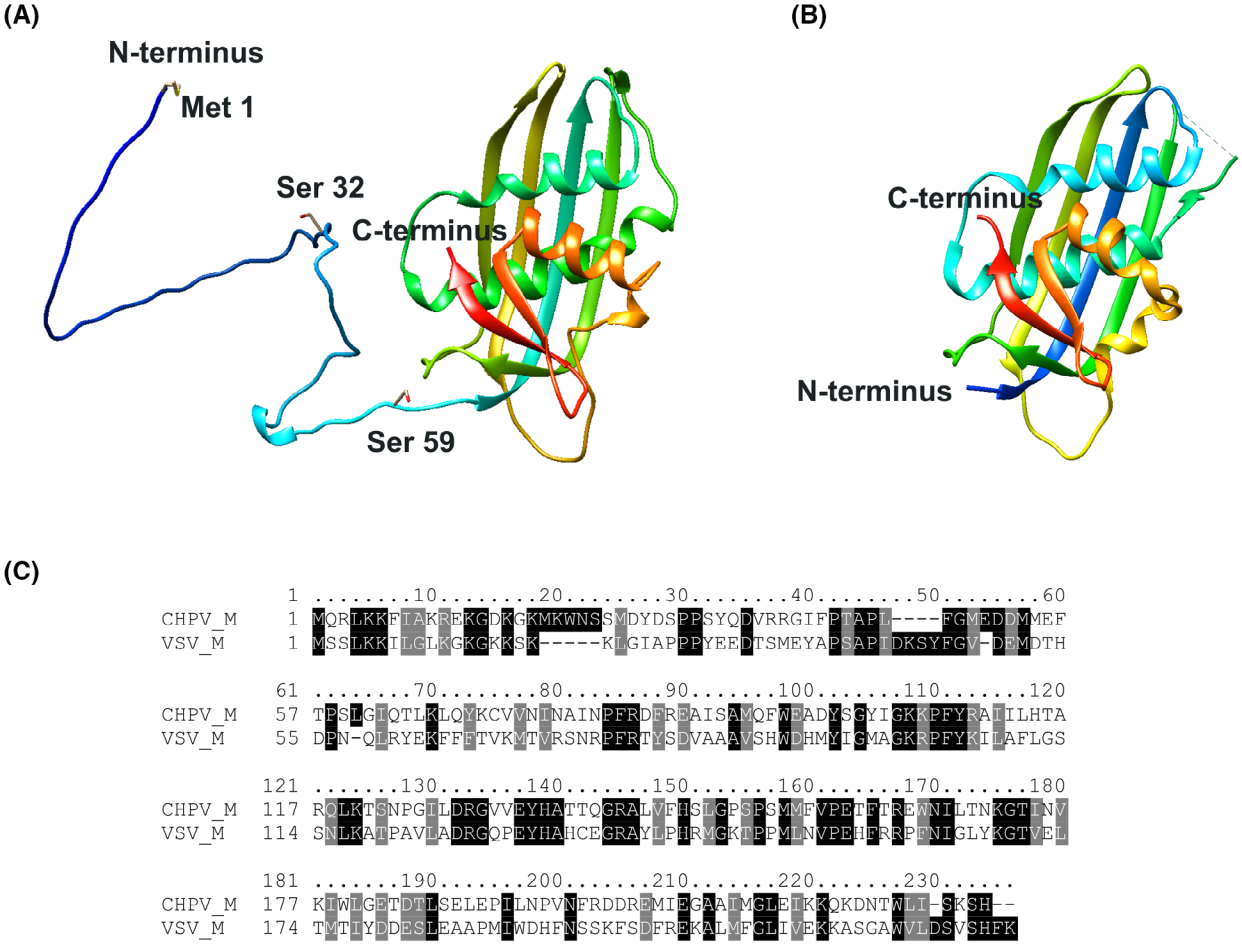
AlphaFold model of CHPV M. (A) Model of CHPV M predicted by AlphaFold within https://www.tamarind.bio/. The overall pLDDT is 76.2. Relevant residues (Met 1, Ser 32, and Ser 59) for construct design are highlighted. (B) Crystal structure of a truncated VSV M (PDB ID 1LG7). (C) Sequence alignment between the CHPV M and the VSIV M protein (Orsay strain). Sequence identity is 29.28%.

AlphaFold [15, 16] within Tamarind Bio (https://www.tamarind.bio/) [17] was used to predict the three‐dimensional structure of full‐length CHPV M from its amino acid sequence, generating five models. The highest‐scoring model was analyzed further (Fig. 1A). Additionally, the Basic Local Alignment Search Tool (BLAST) [18] in UniProt [19] was utilized to identify sequence similarities with other viral M proteins, thereby identifying analogous structures deposited in the PDB [20] (Fig. 1B). Finally, the Clustal Omega program [21] was used to calculate sequence similarities. Sequence alignment was created with T‐Coffee [22, 23] and illustrated with BOXSHADE (Fig. 1C).

### Cloning of CHPV_M, GFP_CHPV_M, GFP_CHPV_32M, and GFP_CHPV_59M for expression in *E. coli*

**Fig. 2:**
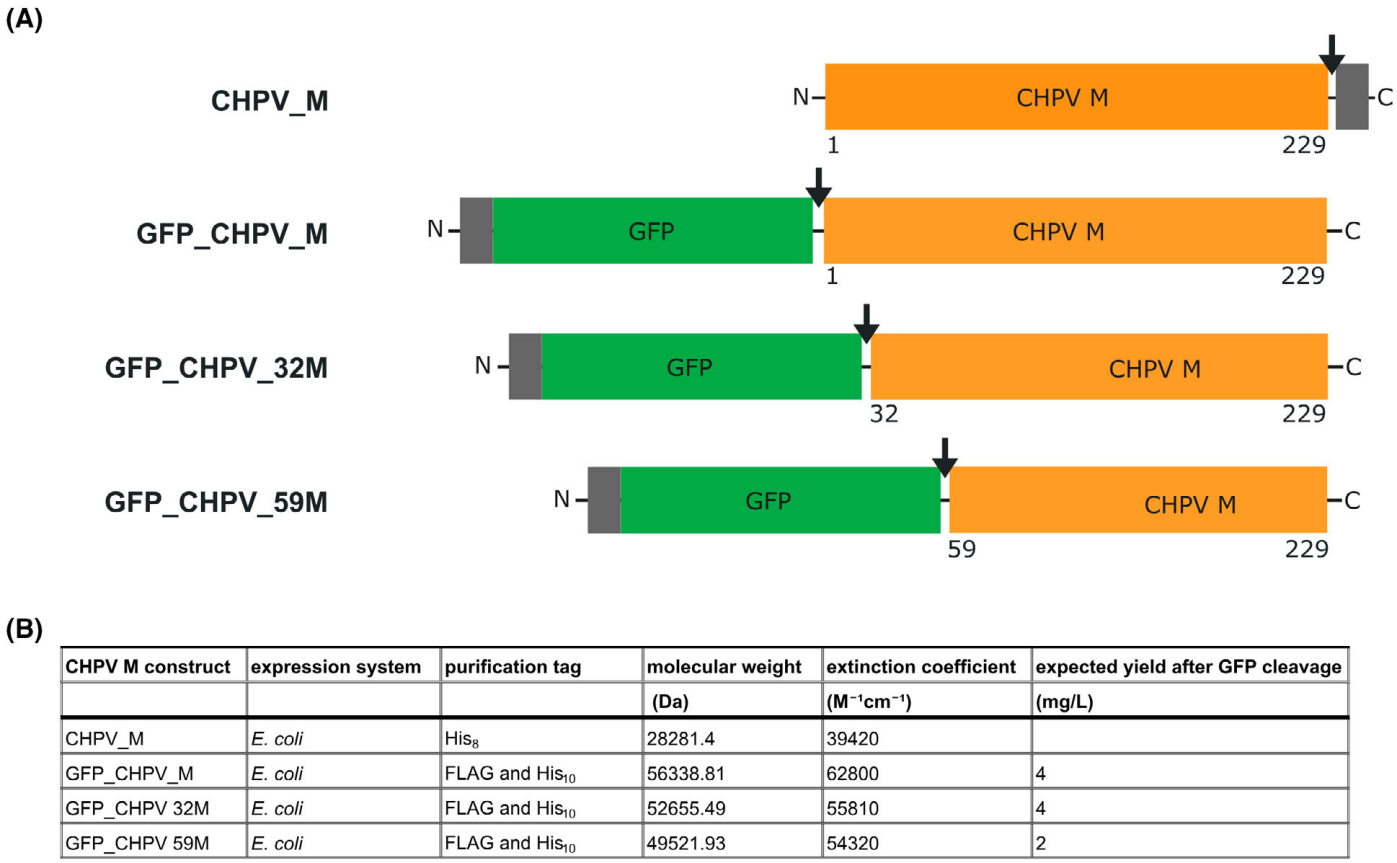
Construct design of CHPV M for expression in *E. coli*. (A) Designed and cloned constructs of CHPV M (Met 1 to His 229, Ser 32 to His 229, and Ser 59 to His 229) are shown as orange boxes. Purification tags are shown as gray boxes. Superfolder GFP is shown as a green box. The TEV protease cleavage sites are marked with black arrows. (B) Table displaying the purification tags and the anticipated yields after GFP cleavage.

A synthetic gene of CHPV M was bought from Twist Bioscience and cloned into the pET28a(+) vector using In‐Fusion Snap Assembly, per the manufacturer's instructions (Tips & Tricks 2). The first construct, termed CHPV_M, includes the complete CHPV M sequence, a TEV protease cleavage site, and a His_8_‐purification tag at the C terminus. In the second cloned construct, the start codon is followed by a FLAG purification tag, a His_10_‐purification tag, the DNA sequence of superfolder GFP, a TEV cleavage site, and the full‐length sequence of CHPV M. This construct was designated GFP_CHPV_M. Additionally, two N‐terminally truncated CHPV M constructs were cloned to reduce intrinsic disorder, starting at serine 32 (GFP_CHPV_32M) or serine 59 (GFP_CHPV_59M) (Fig. 2A,B). All constructs were transformed into Stellar chemically competent cells (Takara Bio, Japan), and the transformants were grown on LB agar plates supplemented with 35 μg·mL^−1^ kanamycin (Tips & Tricks 3).

### Plasmid amplification and purification for transformation of *E. coli* strains

1. Pick a single colony of Stellar chemically competent cells transformed with plasmids of CHPV_M, GFP_CHPV_M, GFP_CHPV_32M, and GFP_CHPV_59M from LB agar plates.
2. Inoculate 5 mL of LB liquid medium with a single colony and grow overnight at 37 °C and 180 r.p.m. in the presence of 35 μg·mL^−1^ kanamycin.
3. Perform plasmid purification using the NucleoSpin Plasmid kit.
4. Confirm the correctness of all constructs through Sanger sequencing.

### Expression screening of CHPV M in *E. coli*

For protein expression screening, the cloned plasmids were transformed into Rosetta™(DE3), Rosetta™(DE3)pLysS, and Rosetta‐gami™ 2(DE3), and the transformants were grown on LB agar plates supplemented with 35 μg·mL^−1^ kanamycin and the *E. coli* strains' specific antibiotics. Initial small‐scale expression screening of CHPV M constructs identified Rosetta‐gami™ 2(DE3) as the most suitable *E. coli* protein expression strain among those tested.

### Large‐scale expression of CHPV M in *E. coli*

1. Transform GFP_CHPV_M, GFP_CHPV_32M, and GFP_CHPV_59M into Rosetta‐gami™ 2 (DE3) and grow transformants on LB agar plates supplemented with 35 μg·mL^−1^ kanamycin.
2. Grow cells from a single colony in LB medium supplemented with 34 μg·mL^−1^ chloramphenicol and 50 μg·mL^−1^ kanamycin at 37 °C and 180 r.p.m. overnight.
3. Add 5 mL starter culture to 2 L TB medium supplemented with 34 μg·mL^−1^ chloramphenicol and 50 μg·mL^−1^ kanamycin at 37 °C and 220 r.p.m. until an OD_600_ of 0.6 is reached, and then cool to 20 °C.
4. Add 1 mm IPTG to induce protein expression.
5. Express protein for 14 h at 20 °C and 220 r.p.m.
6. Harvest the cells by centrifugation (30 min at 4000 ***g*** and 4 °C).
7. Freeze cell pellets (4 g each) in liquid nitrogen and store at −80 °C until purification.

Expression rates of a few milligrams of protein per liter of cell culture (Fig. 2B) were observed for the three constructs fused to a GFP gene on the CHPV M N terminus (GFP_CHPV_M, GFP_CHPV_32M, and GFP_CHPV_59M). The cell pellets collected after centrifugation displayed a strong, visibly vibrant green color, suggesting that the GFP fused to the N terminus minimized the predicted cytopathic effects, which consequently also resulted in increased expression levels of soluble protein. In contrast, CHPV M, which was not fused to GFP, appeared to be cytotoxic.

### Purification of GFP_CHPV_M, GFP_CHPV 32M, and GFP_CHPV 59M

**Fig. 3:**
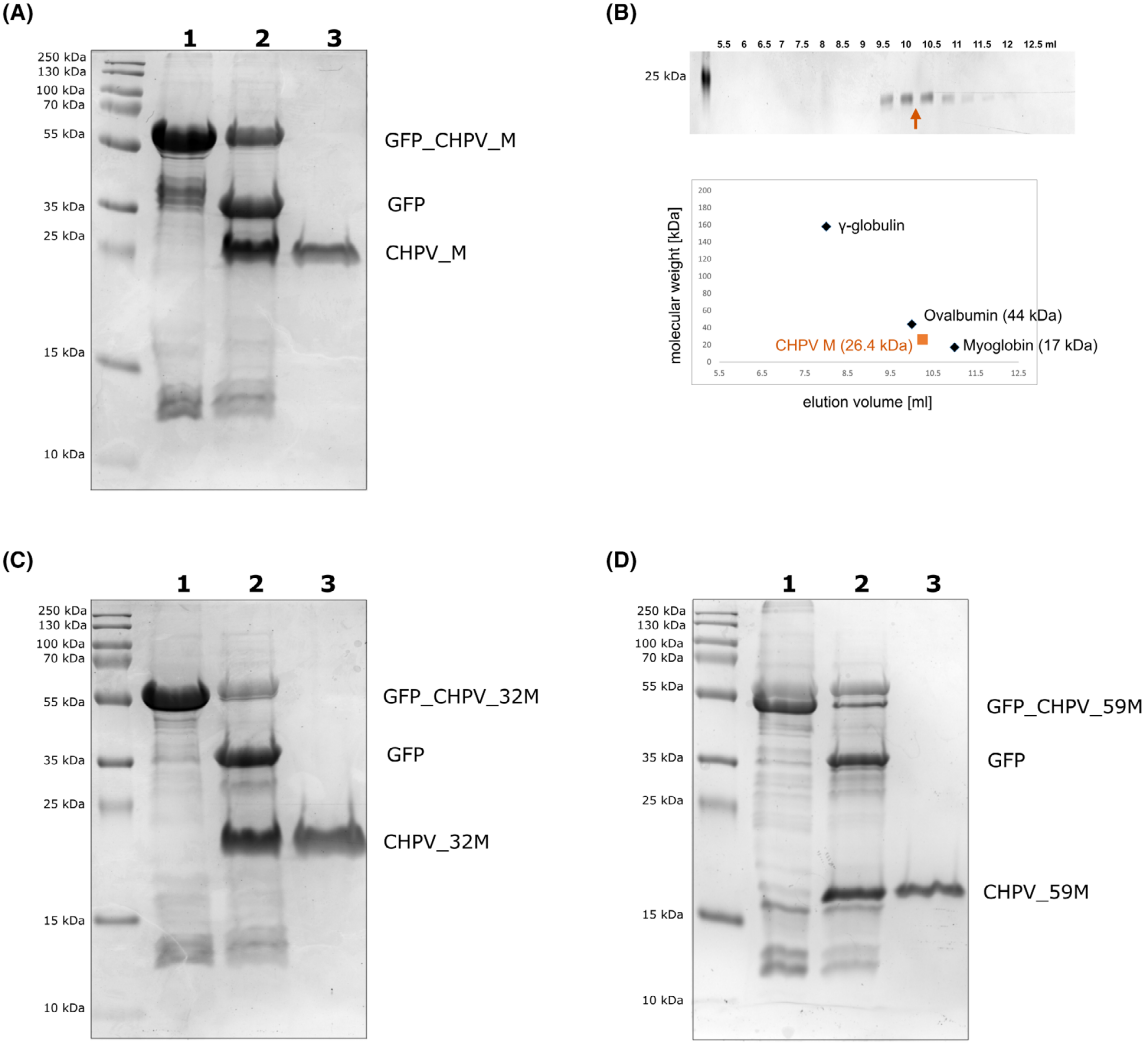
Purification of CHPV M constructs. (A) SDS/PAGE of the CHPV M construct GFP_CHPV_M purified from the *E. coli* protein expression strain Rosetta‐gami™ 2(DE3). Left: protein marker. Lane 1: GFP_CHPV_M (56.3 kDa) eluted from IMAC. Lane 2: GFP_CHPV_M after treatment with the TEV protease for 14 h at 4 °C. Lane 3: purified full‐length CHPV_M containing an additional Serine on the N terminus (26.4 kDa). (B) SDS/PAGE of the purified full‐length CHPV_M construct eluted from size exclusion chromatography. The full‐length CHPV_M (shown as an orange dot) elutes around 10.25 mL between the Biorad gel filtration standards Ovalbumin (44 kDa) and Myoglobin (17 kDa). (C) SDS/PAGE of GFP_CHPV_32M purification. Lane 1: GFP_CHPV_32M (52.6 kDa) eluted from IMAC. Lane 2: GFP_CHPV_32M after treatment with TEV protease for 14 h at 4 °C. Lane 3: purified CHPV_32M (Ser 32 to His 229) containing an additional Serine on the N terminus (22.7 kDa). (D) SDS/PAGE of GFP_CHPV_59M purification. Lane 1: GFP_CHPV_59M (49.5 kDa) eluted from IMAC. Lane 2: GFP_CHPV_59M after treatment with TEV protease for 14 h at 4 °C. Lane 3: purified CHPV_59M (Ser 59 to His 229) containing an additional Serine on the N terminus (19.6 kDa).

Note: The purification was conducted with higher concentrations of NaCl (here 500 mm), as this has been shown to reduce membrane association and the self‐association of other vesiculovirus M proteins [25]. Additionally, 0.01% (w/v) DDM should be added to GFP_CHPV_M and GFP_CHPV 32 M after step 7. This is unnecessary for GFP_CHPV 59 M. The detergent can be removed in subsequent steps.

1. Resuspend one pellet in 50 mL buffer A and lyse using a cooled cell disruptor at 1.5 kbar in two successive rounds (Tips and Tricks 4).
2. Centrifuge the lysate at 45 000 ***g*** for 60 min and 4 °C.
3. Add TALON^®^ metal affinity resin to the supernatant.
4. Allow all the His‐tagged protein to bind to 1 mL TALON resin in a batch format.
5. Transfer the mixture to an empty chromatography column and wash with 3 column volumes of buffer B.
6. Elute with 2 column volumes of buffer C.
7. Perform buffer exchange into buffer D with a G‐25 desalting column, following the manufacturer's instructions.
8. Add TEV protease at a ratio of 1:30 (w/w).
9. Incubate overnight at 4 °C. This results in the CHPV M protein with an additional serine amino acid at the N terminus.
10. Pass the protein solution through a chromatography column containing 1 mL of TALON resin. Collect the flow‐through (Fig. 3A,C,D).
11. Determine the protein concentration in the flow‐through with a spectrophotometer, according to the user guide (https://www.denovix.com/ds‐11‐series‐user‐guide/). MW and ε can be calculated with the ProtParam online tool (https://web.expasy.org/protparam/) [24].

### Thermal shift assay

**Fig. 4:**
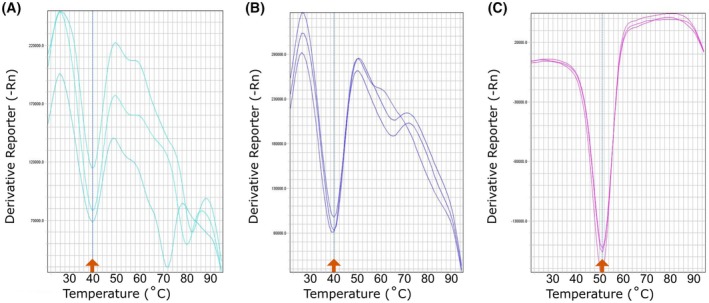
Thermal shift assays. (A) CHPV_M. (B) CHPV_32M. (C) CHPV_59M. Melting temperatures (*T*_m_) are indicated by orange arrows.

1. Prepare samples of purified CHPV_M, CHPV_32M, and CHPV_59M at concentrations of 10 μm.
2. Pipette 30 μL of each construct into MicroAmp Fast Reaction tubes. Prepare each sample in triplicate.
3. Add SYPRO^®^ Orange Protein Gel Stain to the sample just before initiating the measurement. The final concentration is 2× in each tube. Seal the tubes with the caps.
4. Using a real‐time PCR system, record the relative fluorescence units while ramping up the temperature from 20 °C to 95 °C in 1 °C steps (Fig. 4).

### Analytical size exclusion chromatography

An aliquot of the purified CHPV_M was concentrated to 50 μL using a 10 kDa molecular weight cut‐off concentrator to conduct analytical size exclusion chromatography. This concentrated sample was centrifuged for 30 min at 18 000 ***g*** at 4 °C to remove aggregates and then loaded onto a self‐packed Superdex 200 column, which was operated in a cold room at a temperature between 4 and 6 °C. The column's dimensions are 1.5 cm in diameter and 7 cm in height, with an approximate bed volume of 12 mL. Size exclusion chromatography was performed with buffer E. The column's performance was initially assessed by applying gel filtration standards purchased from Bio‐Rad before the concentrated purified CHPV_M was loaded. 500 μL fractions were collected by gravity after sample loading (Fig. 3B).

Analytical size exclusion chromatography of the purified protein, performed at a reduced NaCl concentration of 200 mm, showed that full‐length CHPV M elutes at a volume between the gel filtration standards Ovalbumin (44 kDa) and Myoglobin (17 kDa), corresponding to monomeric CHPV M. Altogether, no self‐association of the full‐length CHPV M protein after GFP cleavage was observed under the conditions outlined here.

### Sodium dodecyl‐sulfate polyacrylamide gel electrophoresis

1. Mix samples with 5× SDS Loading buffer containing DTT.
2. Heat for 10 min at 98 °C.
3. Run 15% SDS/PAGE.
4. Stain gel with Coomassie Brilliant Blue solution (Fig. 3).

### Cloning of CHPV_M, GFP_CHPV_M, and CHPV_M_GFP for expression in mammalian cells

**Fig. 5:**
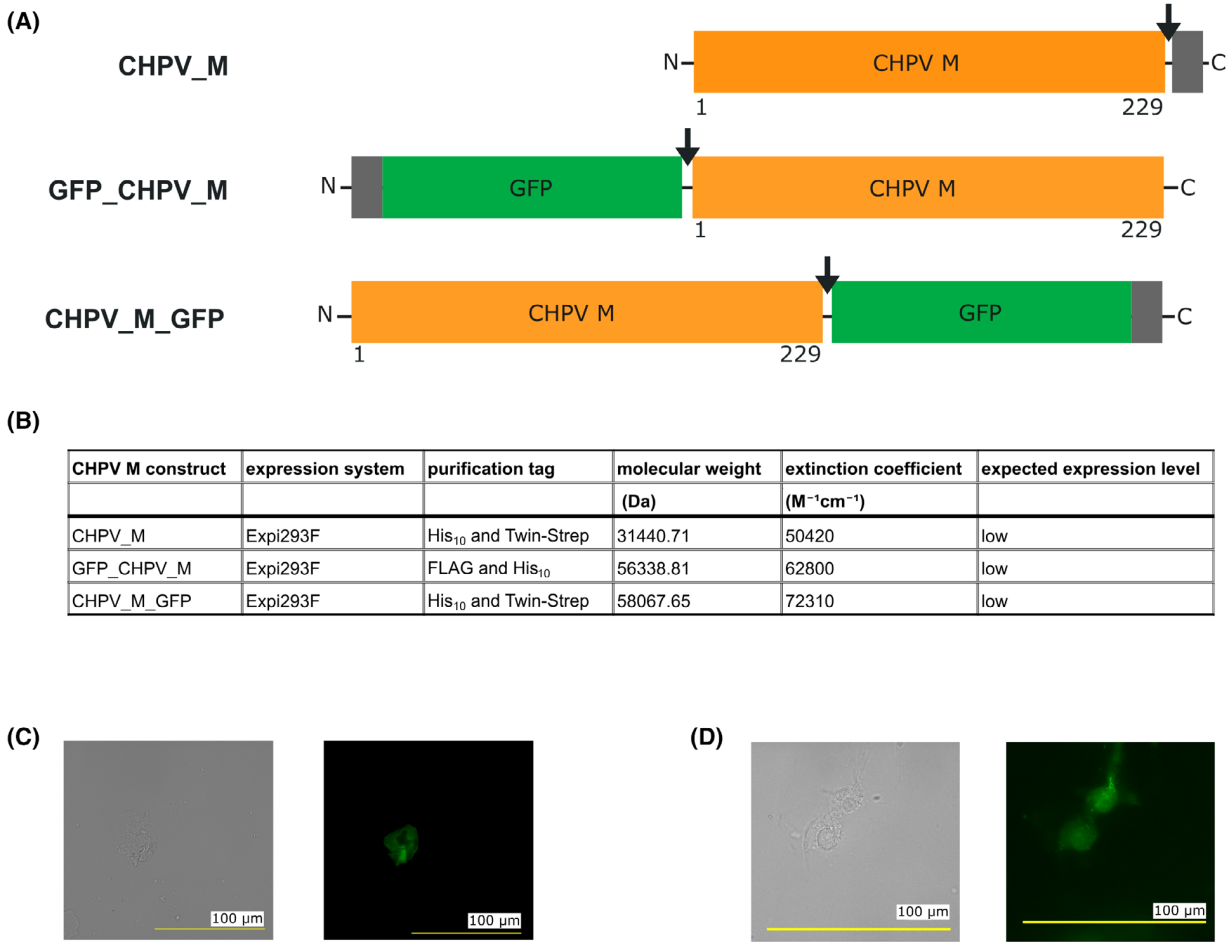
Construct design of CHPV M for expression in mammalian cell lines and fluorescence imaging. (A) Designed and cloned constructs of CHPV M. Full‐length CHPV M is shown as an orange box. Purification tags are shown as gray boxes. Superfolder GFP is shown as a green box. The TEV protease cleavage sites are marked with black arrows. (B) Table displaying the purification tags and the anticipated expression levels of the constructs. (C) Single Expi293F cell transiently transfected with the CHPV_M_GFP construct. (D) Single Expi293F cell transiently transfected with the GFP_CHPV_M construct. The fluorescence of the GFP in the cell is shown (*n* > 3 cells). Scale bar: 100 μm.

The CHPV M gene was also cloned into the pcDNA3.1 vector for expression in mammalian cell lines. In the first construct, termed again CHPV_M, the start codon is followed by the full‐length DNA sequence of CHPV, a TEV cleavage site, a His_10_‐purification tag, and a Twin‐Strep‐tag^®^. In the second construct, the start codon is followed by a FLAG purification tag, a His_10_‐purification tag, the sequence of superfolder GFP, a TEV cleavage site, and the full‐length sequence of CHPV M, identical to GFP_CHPV_M. In the third construct, designated CHPV_M_GFP, the start codon is followed by the full‐length DNA sequence of CHPV M, a TEV cleavage site, the sequence of superfolder GFP, a His_10_‐purification tag, and a Twin‐Strep‐tag^®^ (Fig. 5A,B). All constructs were transformed into Stellar chemically competent cells, and the transformants were grown on LB agar plates supplemented with 100 μg·mL^−1^ ampicillin.

### Plasmid amplification and purification for transient expression in Expi293F

1. Pick a single colony of Stellar chemically competent cells transformed with plasmids of CHPV_M, GFP_CHPV_M, and CHPV_M_GFP from LB agar plates.
2. Inoculate 5 mL of LB liquid medium with a single colony and grow overnight at 37 °C and 180 r.p.m. in the presence of 100 μg·mL^−1^ ampicillin.
3. Perform plasmid purification using the NucleoSpin Plasmid kit.
4. Confirm the correctness of all constructs through Sanger sequencing.
5. Inoculate 100 mL of LB liquid medium with a single colony for endotoxin‐free plasmid purification and grow overnight at 37 °C and 180 r.p.m. in the presence of 100 μg·mL^−1^ ampicillin.
6. Perform plasmid purification using the NucleoBond Xtra Midi EF kit.

### Small‐scale expression screening of CHPV_M and GFP_CHPV_M in Expi293F

Small‐scale expression screening of the constructs CHPV_M and GFP_CHPV_M was performed in Expi293F human cells (Tips & Tricks 5–12). To induce protein expression, 10 mL of Expi293F cell culture (2 × 10^6^ cells·mL^−1^) was transiently transfected with one of the CHPV M plasmids using the ExpiFectamine™ 293 Transfection Kit. Transfected cells were cultured in an orbital shaker at 37 °C or 30 °C and 120 r.p.m. with 8% CO_2_. Cells were harvested 48, 72 h, or 5 days post‐transfection by centrifugation at 900 g for 20 min at 4 °C and then flash‐frozen in liquid nitrogen before storage at −80 °C.

The expression attempts for CHPV_M and GFP_CHPV_M in Expi293F cells resulted in very low expression rates under the tested conditions (not shown). As a result, this expression system is not ideal for structural studies of CHPV M.

However, the expression rates of GFP_CHPV_M and CHPV_M_GFP in Expi293F are adequate for conducting intracellular localization and co‐localization studies with human host proteins using fluorescence microscopy.

### Expression of GFP_CHPV_M and CHPV_M_GFP in mammalian cells

1. Transiently transfected 10 mL of Expi293F cell culture (2 × 10^6^ cells·mL^−1^) with 10 μg pDNA and 30 μL PEI STAR™ at 1 mg·mL^−1^, following the manufacturer's instructions (Tips & Tricks 6).
2. Grow cells in an orbital shaker for 24 h at 37 °C and 120 r.p.m. with 8% CO_2_.

### Fluorescence microscopy

A few cells from a suspension culture were placed on a microscope glass slide and covered with a glass coverslip. Fluorescence was observed using a fluorescence microscope (Keyence, BZ‐X) and a 100× oil immersion objective (Fig. 5C,D).

## Discussion

AlphaFold was utilized to generate a model of the CHPV M protein. The three‐dimensional structure of the full‐length CHPV M (pLDDT: 76.2), as predicted by AlphaFold, shows that the N‐terminal tail of the M protein is likely mostly structurally disordered (residues 1–62), whereas the C‐terminus adopts a globular structure. The model suggests that CHPV M exhibits an overall three‐dimensional structure similar to that of the VSV M crystal (PDB ID 1LG7 [25]) and cryo‐EM (PDB ID 7UML [26]) structures, despite the low sequence similarity of approximately 30%. Both the disordered N‐termini of VSV M and CHPV M are thought to interact with membranes and facilitate self‐association [11]. Meanwhile, the C‐terminal regions of both proteins adopt a comparable globular fold. However, despite the overall structural similarity, the differences in the sequences are likely to impact the interaction of CHPV M with host proteins. Therefore, this virus deserves to be studied independently.

This study presents a protocol for producing full‐length monomeric CHPV M protein and two truncated constructs from *E. coli*, aimed at facilitating structural studies of CHPV M and its complexes with human host proteins.

Initial screening revealed that no significant amounts of soluble CHPV_M could be extracted from *E. coli* or Expi293F cells, indicating a possible membrane association, self‐association, and/or cytotoxicity. Conversely, fusion of a GFP to the N‐terminal disordered tail strongly increased the production of soluble protein in *E. coli*, probably by sterically hindering the CHPV M N terminus from engaging in associations of any kind. The GFP was chosen because it is believed to effectively inhibit membrane association and the self‐association of CHPV M. Its molecular weight of 27 kDa is expected to occupy a volume sufficient to limit steric access to the N terminus. Tags with similar or higher molecular weights are likely to yield comparable results.

Additionally, as vesiculovirus M proteins can be post‐translationally modified in mammalian cells [27], using a bacterial expression system to pursue structural studies is more likely to produce the homogeneous sample required for crystallography and single‐particle cryo‐EM.

From the constructs tested, CHPV_59M is expected to aid in crystallization trials of CHPV M, similar to what was observed with VSV M. Moreover, no self‐association of the purified full‐length CHPV M was detected under the conditions outlined here, and therefore, this construct could also be utilized for crystallization attempts. The production of monomeric full‐length CHPV M also paves the way for conducting protein–protein interaction studies with human host proteins and membranes, where the disordered N‐terminal tail is likely to play a significant role.

Because CHPV M contributes to CHPV‐induced encephalitis by interacting with host proteins in neurons, it is of scientific interest to study the intracellular localization and co‐localization of CHPV M in mammalian cells. For this purpose, a GFP was fused either to the CHPV M C‐terminus or the N terminus. Expression was detected for CHPV_M_GFP and GFP_CHPV_M by fluorescence microscopy, meaning both constructs can be used to study and validate interactions with host proteins in mammalian cell lines.

In conclusion, it is of scientific interest to study the interactions of CHPV M with human host proteins because CHPV is an emerging human pathogen causing deadly encephalitis in children, and no treatment is available to date. CHPV M is expected to induce changes in host cell morphology by interacting with various proteins, ultimately leading to apoptosis. Structural studies of these complexes will aid in understanding the nature of these interactions and in the development of an antiviral drug.

Based upon our findings, the next steps will involve structural studies of CHPV M and complexes of CHPV M with human host proteins, as well as intracellular co‐localization studies of these complexes by fluorescence microscopy. This, however, awaits further investigation.

## Tips and Tricks

1. Chemicals for which no specific supplier was specified can be purchased from any vendor. All chemicals should be of cell culture grade or high purity.
2. The user guide for the In‐Fusion Snap Assembly cloning kit is available here: https://www.takarabio.com/learning‐centers/cloning/in‐fusion‐cloning‐general‐information/in‐fusion‐cloning‐overview.
3. All tubes and flasks for culturing *E. coli* cells must be sterile.
4. All centrifuges and buffers used for protein purification should be pre‐cooled to 4 °C, and the purification should be performed at 4 °C.
5. The user guide that comes with the Expi293F cell vial is available here: https://www.thermofisher.com/order/catalog/product/de/en/A14527.
6. The user guides for the PEI STAR™ transfection reagent are available here: https://www.tocris.com/products/pei‐transfection‐reagent_7854.
7. Researchers must follow laboratory rules and safety guidelines when handling Expi293F cells (https://www.thermofisher.com/de/en/home/references/gibco‐cell‐culture‐basics.html).
8. Expi293F cells in suspension cultures should be maintained between 0.2 x10^6^ cells·mL^−1^ and 2 × 10^6^ cells·mL^−1^ in FreeStyle™ 293 Expression Medium at 37 °C and 8% CO_2_ on a shake platform set to 120 r.p.m..
9. All flasks for culturing Expi293F cells must be sterile and endotoxin‐free.
10. The Expi293F cell culture volume should not exceed 20% of the flask's total volume to allow for aeration and gas exchange.
11. Cell viability at the time of transient transfection should be greater than 95%.
12. Transient transfections must be carried out under sterile conditions and require the use of a laminar flow hood.

## Conflict of interest

The author declares that she has no known competing financial interests or personal relationships that could have appeared to influence the work reported in this paper.

## Author contributions

MG designed and conducted research, analyzed data, and wrote the paper.

## Data Availability

Data will be made available upon request.
